# Insulin resistance is associated with reductions in specific cognitive domains and increases in CSF tau in cognitively normal adults

**DOI:** 10.1038/s41598-017-09577-4

**Published:** 2017-08-29

**Authors:** Simon M. Laws, Scott Gaskin, Amy Woodfield, Velandai Srikanth, David Bruce, Paul E. Fraser, Tenielle Porter, Philip Newsholme, Nadeeja Wijesekara, Samantha Burnham, Vincent Doré, Qiao-Xin Li, Paul Maruff, Colin L. Masters, Stephanie Rainey-Smith, Christopher C. Rowe, Olivier Salvado, Victor L. Villemagne, Ralph N. Martins, Giuseppe Verdile

**Affiliations:** 1 Collaborative Genomics Group, Centre of Excellence for Alzheimer’s Disease Research and Care, School of Medical and Health Sciences, Edith Cowan University, Joondalup, Western Australia Australia; 2 School of Biomedical Sciences, Faculty of Health Sciences, Curtin Health Innovation Research Institute, Curtin University, Perth, Western Australia Australia; 3 Cooperative Research Centre for Mental Health, Melbourne, Australia; 4 Peninsula Medical School, Monash University, Melbourne, Australia; 5 School of Medicine and Pharmacology, University of Western Australia, Perth, Australia; 6 Tanz Centre for Research in Neurodegenerative Diseases, University of Toronto, Toronto, ON Canada; 7 Department of Medical Biophysics, University of Toronto, Toronto, ON Canada; 8 Centre of Excellence for Alzheimer’s Disease Research and Care, School of Medical and Health Sciences, Edith Cowan University, Joondalup, Western Australia Australia; 9 eHealth, CSIRO Health and Biosecurity, Parkville, Vic Australia; 10 eHealth, CSIRO Health and Biosecurity, Herston, QLD Australia; 11 Department of Nuclear Medicine and Centre for PET, Austin Health, Heidelberg, VIC Australia; 12 The Florey Institute of Neuroscience and Mental Health, The University of Melbourne, Parkville, Victoria Australia; 13 CogState Ltd., Melbourne, Victoria Australia; 14 Department of Medicine, Austin Health, The University of Melbourne, Heidelberg, Victoria Australia; 15 Biomedical Sciences, Faculty of Medicine and Health Sciences, Macquarie University, New South Wales, Australia

## Abstract

Growing evidence supports the hypothesis that type 2 diabetes (T2D) increases the risk of developing dementia. Experimental evidence from mouse models demonstrates that the induction of T2D/insulin resistance (IR) can promote the accumulation of Alzheimer’s disease (AD) pathological features. However, the association of T2D with pathological and clinical phenotypes in humans is unclear. Here we investigate the relationship of indices of IR (HOMA-IR) and pancreatic β-cell function (HOMA-B) with cognitive performance across several domains (Verbal/Visual Episodic Memory, Executive Function, Language and a measure of Global cognition) and AD biomarkers (CSF Aβ42, T-tau/P-tau, hippocampal volume and neocortical Aβ-amyloid burden). We reveal that HOMA-IR (p < 0.001) incrementally increases across diagnostic groups, becoming significantly elevated in the AD group compared with cognitively normal (CN) adults. In CN adults, higher HOMA-IR was associated with poorer performance on measures of verbal episodic memory (p = 0.010), executive function (p = 0.046) and global cognition (p = 0.007), as well as with higher CSF T-tau (p = 0.008) and P-tau (p = 0.014) levels. No association was observed with CSF Aβ or imaging modalities. Together our data suggest that IR may contribute to reduced cognitive performance and the accumulation of CSF tau biomarkers in cognitively normal adults.

## Introduction

Epidemiological studies indicate that Type 2 diabetes (T2D) is associated with an increased risk of dementia^1–5^. Clinical studies using cross-sectional designs support this association by showing that cognition is worse in patients with T2D as compared to matched controls without T2D^6, 7^. Furthermore, studies of structural magnetic resonance imaging (MRI) show that in T2D cognitive impairment is associated with greater levels of vascular lesions as well as with brain atrophy^6–10^. Prospective MRI studies also show that in T2D brain atrophy occurs at faster rates than in normal ageing^11, 12^, suggesting that T2D accelerates neurodegeneration.

Animal studies provide additional evidence to show that inducing T2D/insulin resistance (IR) can promote the pathological changes characteristic of Alzheimer’s disease (AD), specifically accumulation of amyloid-beta (Aβ) and tau (see review ref. 13). These studies also implicate common inflammatory or oxidative stress pathways that link these two chronic diseases of ageing (see review ref. 14). However, the association between cognition and AD pathology in human studies and the stage of AD progression where IR has greater impact remains unclear. Evidence to date using Positron emission tomography (PET) studies have so far been inconclusive.

In AD, PET studies of cerebral glucose metabolism (18F-deoxyglucose PET: FDG-PET) and Aβ deposition (e.g. C^11^-Pittsburg compound B-PET: PiB-PET) show that reduced neuronal glucose metabolism and increased levels of neocortical Aβ accumulation are features that occur early in the disease (see review ref. 15). A small number of cohort studies have investigated the relationship between these imaging markers of AD and T2D. A cross-sectional study in the population-based Mayo Clinic Study of Ageing assessed cerebral glucose metabolism (FDG-PET) and Aβ deposition (PiB-PET) in healthy older and cognitively normal (CN) adults and older people with T2D. The study showed that compared to the controls, those with T2D displayed cerebral hypometabolism, particularly in those regions severely affected in AD, but no differences in neocortical amyloid load^16^. In the Baltimore, longitudinal study of Ageing (BLSA), no association was observed between measures of peripheral IR or glucose tolerance and neocortical Aβ load (in a PiB-PET scanned cohort) or other pathological features of AD in post-mortem brain^17^. More recently in the Alzheimer’s Disease Neuroimaging Initiative (ADNI) study, no association was shown betweenT2D and accumulation of neocortical Aβ load (PiB-PET) or increases in CSF Aβ42^10^. Instead, T2D was associated with lower cortical thickness an increase in CSF total tau (T-Tau) and phosphorylated tau (P-tau). Together, these findings suggest that IR/T2D is not associated with cerebral accumulation of Aβ but with other hallmarks of the disease. However, in a recent cross-sectional study, Aβ deposition was associated with a higher Homeostatic Model Assessment of IR (HOMA-IR) in late-middle aged, normoglycaemic cognitively normal participants^18^. The conflicting results in the current literature may be in part due to demographic differences in populations (e.g. age, clinical staging of disease and disease progression) and study design. Further, recent meta-analyses suggest that sex can also mediate T2D associations with dementias and associated co-morbidities, such as stroke^19–21^. Thus, the relationship between IR and clinical and pathological features of the early stages of AD, and sex specific effects, requires further study, particularly in normoglycaemic individuals and prior to the onset of cognitive impairment.

In addition to IR, β-cell hyperactivity and dysfunction and subsequent hyperinsulinemia also contribute to hyperglycaemia and T2D (see review ref. 22). Further, recent evidence indicates β-cell dysfunction in AD rodent models^23, 24^ and that Aβ and Tau have been shown to accumulate in human post-mortem pancreatic tissue in T2D^25^, possibly contributing to β-cell dysfunction. Despite this evidence, there is a lack of literature investigating pancreatic β-cell activity (HOMA-B) on cognition and AD related pathology.

The overall aim of this study was to investigate if assessments of IR (HOMA-IR) or pancreatic β-cell function (HOMA-B) are altered across diagnostic groups and ascertain their associations with pathological and clinical expressions of AD in the well characterised Australian Imaging Biomarker and Lifestyle (AIBL) study. We hypothesised that HOMA-IR and HOMA-B are altered across diagnostic groups and are associated with poorer cognitive performance and higher burdens of neuroimaging/CSF biomarker load in cognitively normal participants.

## Results

Clinical and cognitive descriptive data for the diagnostic groups [cognitively normal adults (CN), mild cognitive impairment (MCI) and AD] are presented in Tables 1 and 2, respectively. A significant difference in age and frequency of the Apolipoprotein E (*APOE*) ε4 allele was observed across all diagnostic groups (Table 1), with AD and MCI being older and having a significantly higher *APOE* ε4 frequency compared to CN (p < 0.001). Significant differences between groups for pathological features and cognitive measures confirmed a clear differentiation between diagnostic groups (Table 2).

**Table 1:** Baseline demographic and clinical pathology data for clinical classifications across the cohort. All data is presented as means ± standard deviations or %, where indicated. CN, cognitive normal; MCI, Mild cognitively impaired; AD, Alzheimer’s disease; ^†^HOMA indices underwent Box-Cox transformations prior to analysis.

	CN	MCI	AD	P value
n	905	156	203	
Age (years)	70.6 ± 6.8	75.1 ± 7.6	76.4 ± 7.7	<0.001
Sex (Female, %)	59.7	53.2	56.9	0.282
*APOE* ε4 (Positive, %)	26.8	53.2	63.7	<0.001
BMI	26.6 ± 4.1	25.5 ± 4.1	25.1 ± 4.4	<0.001
Diabetes (%)	7.8	7.7	9.3	0.772
Diabetic Medication (%)	4.9	6.4	6.9	0.431
Hypertension (%)	34.1	36.8	26.5	0.067
Smoking (%)	4.0	1.9	1.0	0.056
Insulin (mU/L) *(Reference Range* < *12)*	7.42 ± 7.16	8.88 ± 12.2	8.12 ± 7.0	0.074
Glucose (mmol/L) *(Reference range 3*.*0–5*.*4)*	5.19 ± 0.79	5.20 ± 0.99	5.39 ± 1.30	0.014
HOMA-IR^†^	0.617 ± 0.189	0.635 ± 0.198	0.647 ± 0.191	0.091
HOMA-B^†^	4.73 ± 0.80	4.83 ± 0.90	4.76 ± 0.922	0.302

**Table 2:** Baseline AD-related phenotypic data and cognitive descriptive statistics for clinical classifications across the cohort. All data is presented as means ± standard deviations. CN, cognitive normal; MCI, Mild cognitively impaired; AD, Alzheimer’s disease; VEM, Verbal Episodic Memory; ViEM, Visual Episodic Memory; EF, Executive Function; LANG, Language; GLOBAL, Global cognitive composite; HV, Intracranial volume corrected Hippocampal Volume; NAB, Neocortical Amyloid Burden; SUVR-BeCKeT, a transformation of native SUVR into PiB-like SUVR.

	CN	MCI	AD	P value
**Cognitive Composites**				
VEM	0.032 ± 1.20 (n = 891)	−2.69 ± 1.49 (n = 146)	−3.93 ± 1.70 (n = 152)	<0.001
ViEM	−0.010 ± 1.31 (n = 900)	−2.02 ± 1.39 (n = 152)	−4.42 ± 1.36 (n = 166)	<0.001
EF	0.006 ± 1.08 (n = 883)	−1.24 ± 1.23 (n = 146)	−3.37 ± 1.89 (n = 139)	<0.001
LANG	0.015 ± 0.72 (n = 887)	−1.26 ± 0.90 (n = 153)	−3.50 ± 1.32 (n = 174)	<0.001
GLOBAL	0.026 ± 1.17 (n = 889)	−3.07 ± 1.68 (n = 146)	−6.37 ± 2.14 (n = 151)	<0.001
**Neuroimaging/CSF Biomarkers**				
HV (cm^3^)	2.93 ± 0.27 (n = 229)	2.62 ± 0.42 (n = 52)	2.39 ± 0.38 (n = 38)	<0.001
NAB (SUVR-BeCKeT)	1.38 ± 0.40 (n = 262)	1.86 ± 0.53 (n = 69)	2.11 ± 0.50 (n = 48)	<0.001
CSF Aβ42	681.2 ± 201.9 (n = 36)	590.3 ± 229.7 (n = 14)	496.9 ± 243.5 (n = 16)	0.022
CSF T-tau	330.6 ± 236.8 (n = 36)	512.0 ± 321.0 (n = 14)	587.5 ± 235.4 (n = 16)	0.003
CSF P-tau	58.2 ± 29.1 (n = 36)	75.4 ± 34.9 (n = 14)	79.6 ± 23.7 (n = 16)	0.031
CSF Aβ42:T-tau	2.72 ± 1.40 (n = 36)	1.77 ± 1.30 (n = 14)	1.01 ± 0.79 (n = 16)	<0.001
CSF Aβ42:P-tau	13.82 ± 6.12 (n = 36)	10.35 ± 7.04 (n = 14)	6.85 ± 4.46 (n = 16)	0.001

### Relationships between T2D markers and clinical diagnosis

**Figure 1:**
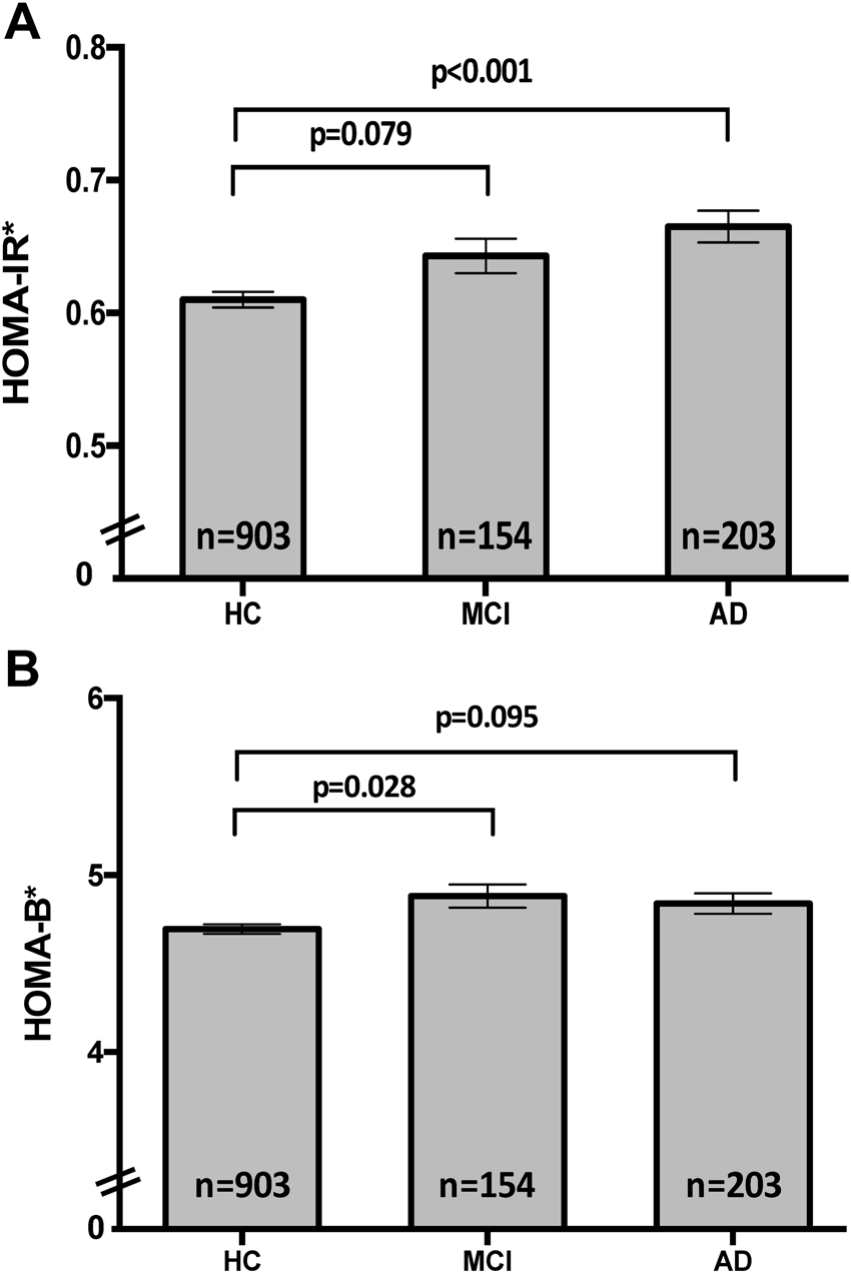
HOMA-IR (**A**) and HOMA-B (**B**) at baseline within the clinical classifications of AIBL: *HOMA-IR and HOMA-B represented as Estimated Marginal Means ( ± SEM) of Box-Cox transformed raw data. Univariate analysis was performed covarying for BMI, sex, %diabetes, %diabetes medication, %hypertension, smoking, age and *APOE* ε4 (HOMA-IR, F = 8.656, p < 0.001; HOMA-B, F = 4.564, p = 0.011). Presented p-values are calculated using Post-hoc Bonferoni analysis.

Serum glucose levels were increased significantly in AD and MCI compared with CN (p = 0.014) (AD > MCI > CN), and a non-significant trend was observed for serum insulin HOMA-IR, but not for HOMA-B (Table 1). These group differences in the HOMA indices became statistically significant after co-varying for BMI, sex, diabetes, use of diabetes medication, smoking, age and *APOE* genotype. HOMA-IR (Fig. 1A) was significantly increased across diagnostic groups (ANCOVA, F = 8.656, p < 0.001) with post-hoc analysis showing a significant increase in HOMA-IR in the AD group (Bonferroni p < 0.001) compared to CN. A clinical group difference was also observed for HOMA-B (Fig. 1B; F = 4.564, p = 0.011), though between the MCI and CN groups (Bonferroni p = 0.028). In both cases, significant differences were only observed in females (HOMA-IR, F = 6.989 p = 0.001; HOMA-B, F = 5.575 p = 0.004) but not males (HOMA-IR, F = 2.603 p = 0.075; HOMA-B, F = 0.911 p = 0.403). In females HOMA-IR was still only observed to be different in the AD group (Bonferroni p = 0.001) compared to CN, whilst HOMA-B, was significantly lower in CN compared to both the MCI (Bonferroni p = 0.041) and AD (Bonferroni p = 0.017) groups.

### HOMA-IR is associated with cognitive performance and CSF biomarkers

A significant inverse relationships were observed (Table 3) between HOMA-IR and the cognitive composites, verbal episodic memory (β = −0.65, p = 0.010), executive functioning (β = −0.48, p = 0.046) and global composite (β = −0.68, p = 0.007). Significant positive relationships were also observed with CSF T-tau (β = 830.2, p = 0.008) and P-tau (β = 95.9, p = 0.014). Increases in HOMA-B were only observed to be associated with reductions in executive functioning (β = −0.095, p = 0.044) and the global composite (β = −0.011, p = 0.043). Stratification by sex (Table 4, female; Table 5

**Table 3:** Relationship between HOMA indices and cognitive composites and neuroimaging/CSF biomarkers in cognitively normal older adults.

**Cognitive Composites**														
	**VEM**		**ViEM**		**EF**		**LANG**		**Global**					
	**β**	**Sig**	**β**	**Sig**	**β**	**Sig**	**β**	**Sig**	**β**	**Sig**				
**HOMA-IR**	−0.65	***0.010***	−0.18	0.527	−0.467	***0.046***	−0.21	0.180	−0.68	***0.007***				
**HOMA-B**	−0.09	0.093	−0.04	0.451	−0.10	***0.044***	−0.02	0.459	−0.11	***0.043***				
**Neuroimaging/CSF Biomarkers**														
	**NAB**		**HV**		**CSF T-tau**		**CSF P-tau**		**CSF Aβ42**		**CSF Aβ42:T-tau**		**CSF Aβ42:P-tau**	
	**β**	**Sig**	**β**	**Sig**	**β**	**Sig**	**β**	**Sig**	**β**	**Sig**	**β**	**Sig**	**β**	**Sig**
**HOMA-IR**	0.06	0.691	−0.05	0.542	830.2	***0.008***	95.9	***0.014***	−99.2	0.712	−3.30	0.066	−11.9	0.128
**HOMA-B**	−0.02	0.490	0.01	0.538	125.3	0.08	155.7	0.08	9.63	0.871	−0.52	0.193	−2.05	0.243

Linear regression analysis covarying for (in all dependent variables) *APOE* ε4, BMI, %Diabetes, %Diabetes medication, %Hypertension and smoker and (for biomarkers only) sex and age. VEM, Verbal Episodic Memory; ViEM, Visual Episodic Memory; EF, Executive Function; LANG, Language; GLOBAL, Global cognitive composite. HV, Intracranial volume corrected Hippocampal Volume; NAB, Neocortical Amyloid Burden; CSF, Cerebrospinal Fluid.

**Table 4:** Relationship between HOMA indices and cognitive composites and neuroimaging/CSF biomarkers in cognitively normal older adults (FEMALES).

**Cognitive Composites**														
	**VEM**		**ViEM**		**EF**		**LANG**		**Global**					
	**β**	**Sig**	**β**	**Sig**	**β**	**Sig**	**β**	**Sig**	**β**	**Sig**				
**HOMA-IR**	−0.63	***0.046***	−0.45	0.220	−0.61	***0.042***	−0.25	0.218	−0.79	***0.014***				
**HOMA-B**	−0.08	0.222	−0.07	0.367	−0.10	0.134	−0.01	0.800	−0.11	0.111				
**Neuroimaging/CSF Biomarkers**														
	**NAB**		**HV**		**CSF T-tau**		**CSF P-tau**		**CSF Aβ42**		**CSF Aβ42:T-tau**		**CSF Aβ42:P-tau**	
	**β**	**Sig**	**β**	Sig*	**β**	**Sig**	**β**	**Sig**	**β**	**Sig**	**β**	**Sig**	**β**	**Sig**
**HOMA-IR**	0.09	0.570	−0. 16	0.155	687.0	***0.023***	93.4	***0.031***	−107.8	0.804	−4.64	0.094	−17.4	0.131
**HOMA-B**	0.01	0.666	−0.02	0.480	136.4	0.056	19.4	0.055	50.2	0.613	−0.65	0.325	−2.44	0.369

Linear regression analysis covarying for (in all dependent variables) *APOE* ε4, BMI, %Diabetes, %Diabetes medication, %Hypertension and smoker and (for biomarkers only) age. VEM, Verbal Episodic Memory; ViEM, Visual Episodic Memory; EF, Executive Function; LANG, Language; GLOBAL, Global cognitive composite. HV, Intracranial volume corrected Hippocampal Volume; NAB, Neocortical Amyloid Burden; CSF, Cerebrospinal Fluid.

**Table 5:** Relationship between HOMA indices and cognitive composites and neuroimaging/CSF biomarkers in cognitively normal older adults (MALES).

**Cognitive Composites**														
	**VEM**		**ViEM**		**EF**		**LANG**		**Global**					
	**β**	**Sig**	**β**	**Sig**	**β**	**Sig**	**β**	**Sig**	**β**	**Sig**				
**HOMA-IR**	−0.63	0.133	0.15	0.731	−0.28	0.452	−0.13	0.601	−0.48	0.252				
**HOMA-B**	−0.16	0.057	0.05	0.579	−0.09	0.223	−0.06	0.241	−0.16	0.060				
**Neuroimaging/CSF Biomarkers**														
	**NAB**		**HV**		**CSF T-tau**		**CSF P-tau**		**CSF Aβ42**		**CSF Aβ42:T-tau**		**CSF Aβ42:P-tau**	
	**β**	**Sig**	**β**	**Sig**	**β**	**Sig**	**β**	**Sig**	**β**	**Sig**	**β**	**Sig**	**β**	**Sig**
**HOMA-IR**	0.00	0.999	0.15	0.227	857.8	0.280	82.0	0.387	−333.0	0.369	−2.53	0.387	−10.45	0.413
**HOMA-B**	−0.07	0.191	0.04	0.06	134.7	0.448	13.64	0.515	−79.0	0.328	−0.41	0.530	−1.97	0.540

Linear regression analysis covarying for (in all dependent variables) *APOE* ε4, BMI, %Diabetes, %Diabetes medication, %Hypertension and smoker and (for biomarkers only) age. VEM, Verbal Episodic Memory; ViEM, Visual Episodic Memory; EF, Executive Function; LANG, Language; GLOBAL, Global cognitive composite. HV, Intracranial volume corrected Hippocampal Volume; NAB, Neocortical Amyloid Burden; CSF, Cerebrospinal Fluid.

, male) revealed that prior significant associations held between HOMA-IR and verbal episodic memory (β = −0.63, p = 0.046), executive function (β = −0.61, p = 0.042), the global cognitive composite (β = −0.79, p = 0.014) and both CSF T-tau (β = 639.5, p = 0.048) and P-Tau (β = 93.4, p = 0.031) in females. No significant associations were observed for either HOMA-IR or HOMA-B in males.

## Discussion

Our findings demonstrate modest yet significant differences, in both HOMA-IR and HOMA-B, between the clinical classifications of AD, MCI and CN within the AIBL cohort, after covarying for potential confounding variables. Within clinical classifications, compared to the CN group, the AD group had higher HOMA-IR. These findings are consistent with previous reports showing that the prevalence of IR is greater in MCI/AD patients than controls^26–28^. However, in the current study HOMA-B was increased only in the MCI group, suggesting an increase in β-cell function/insulin secretion in this group, an observation consistent with the observed trend towards increasing absolute insulin levels across groups. This may represent a response to control increasing glucose levels during disease progression^29^, which was also observed in this study. Overall, these initial findings suggested that increased β -cell function/insulin secretion is associated with cognitive impairment, at least in non-demented adults. However, in the absence of overt cognitive impairment (i.e. CN group), compared to HOMA-IR, HOMA-B had no or weak associations with functioning of cognitive domains. This suggests that changes in insulin sensitivity is the stronger, earlier contributor to impairments in cognition. Longitudinal analysis in the cognitively normal that do or do not show clinical disease progression may provide further support for this notion.

We also observed sex differences in levels of HOMA-IR and HOMA-B between diagnostic groups, where upon stratification by sex, the increases observed in the MCI or AD groups were only observed in females. These findings are consistent with outcomes from meta-analyses which indicate women with T2D are at higher risk of stroke and dementia compared to men with T2D^19–21^ and may be a consequence of several factors. For example, studies in different ethnic groups have suggested that age related increases in the prevalence of metabolic syndrome are greater in women then in men (see review ref. 30). Similarly, impaired glucose tolerance has been reported to be more prevalent in older women than men, although impaired fasting glycaemia more prevalent in men^31^. Further, changes in hormonal status also contribute to an age-associated increased prevalence of metabolic syndrome in women^32^ and may be driven by androgen/oestrogen imbalances during the peri-menopausal period^33^. For example reduction in the neuroprotective effects of oestrogen or increases in gonadotropins may act to promote AD related pathological changes (see review ref. 34). With longitudinal study, the interactions between biological markers of IR and indices of AD related biology, and its clinical expression, would provide evidence to confirm our findings that these differences are stronger in woman than men, whilst also providing an indication of the potential contributions of hormones.

Having shown that increases in IR were associated with clinical stages of AD, we wished to ascertain the associations of HOMA-IR and HOMA-B with pathological and clinical expressions of AD prior to the onset of clinical cognitive impairment. In the CN group, greater HOMA-IR was associated with reductions in global cognition and in two of the four cognitive domains assessed, namely verbal episodic memory and executive functioning. A weaker association was observed between HOMA-B and executive function and global cognition. Our findings are in agreement with previous studies showing similar associations of cognitive functioning with HOMA-IR in cognitively normal individuals from community-based volunteer^35^, population based^36^ or family based^37^ studies. Episodic memory is one of the earliest cognitive domains that show changes in the pre-clinical stages of AD. Previous studies have observed episodic memory changes occurring 4–8 years prior to executive function and up to 10-years prior to changes in other cognitive domains^38–40^. Compared to non-diabetics, verbal memory and executive functioning are also strongly affected in T2D^41, 42^. The association of IR with episodic memory suggests that it plays a role at early preclinical stages of the disease process.

Stratification by sex revealed these associations were again present in females only. In previous studies, there have been varied findings with respect to the effect of sex on the association between HOMA-IR and cognitive functioning. In a cross-sectional study of cognitively healthy elderly community volunteers, higher HOMA-IR was associated with lower verbal fluency performance and reduced grey matter in the temporal lobe of both men and women^35^. However, in a large, family based Dutch study of 1898 participants, higher HOMA-IR was associated with poorer executive functioning in women only^37^. A similar association with verbal fluency in women only was shown recently in a population based study of ~6000 adults^36^. This association was independent of a marker of glucose levels (glycated haemoglobin; HbA1c), indicating that IR may have early effects on cognition and maybe detected prior to development of significant hyperglycaemia and T2D^36^. Further, in the same study HOMA-IR was associated with poorer verbal fluency in *APOE* ε4 non-carriers only. Several factors could account for these mixed results including age, cohort characteristics (family vs population vs community based) and the size of the cohort. For example, those studies that show associations in women only^36, 37^ the analysis was undertaken in larger family or population based cohorts or were younger (~45 yrs. and 53 yrs., respectively) compared to our study and that of Benedict and colleagues^35^ (71 and 73 yrs., respectively).

IR was also associated with biomarkers of AD pathology within the cognitively normal group where increases in HOMA-IR were associated with increases in CSF T-tau and P-tau. No such relationships were observed with neocortical Aβ burden or CSF-Aβ42 in these groups. These findings suggest that, at earlier stages of the disease process there is a stronger relationship with tau rather than Aβ. Cross-sectional cohort studies evaluating imaging modalities (structural MRI, PET) or AD biomarkers have shown similar results. In these studies, lower cortical thickness and cerebral hypometabolism, but not changes in Aβ were associated with T2D^8, 16, 17^. More recently, in the ADNI study, T2D-related reduction in cortical thickness was associated with increases in CSF P-tau, but not with neocortical Aβ burden or CSF Aβ42, across diagnostic groups of CN, MCI, and AD^10^. Our study was not powered to similarly stratify for T2D as only a small proportion (~8%) of the total cohort had the condition. The association of HOMA-IR with increases in CSF tau, even after controlling for T2D, suggests that IR is an early contributor to this process. However, it is acknowledged that this is a cross-sectional analysis and a longitudinal follow-up is required to further establish IR as a determinant of cognitive decline and accumulation of AD pathological features.

Numerous studies in various animal models have shown that diet (HF diet/sucrose/fructose) or chemical (administration of the β-cell toxin streptozotocin (STZ)) induced diabetes promote, tau phosphorylation, and synaptic/neurodegeneration^23, 43–47^. The potential mechanisms may involve neuroinflammatory and oxidative stress mechanisms^14^. In addition, impaired cerebral insulin signalling, promoted by IR and T2D, plays a key role in this process and in triggering tau hyper phosphorylation through increased activity of GSK-3β, a major protein kinase that phosphorylates tau (see review ref. 13). These *in vivo*, animal studies also support a role for T2D/IR in promoting Aβ build up in the brain, where availability of Aβ degrading enzymes such as the insulin degrading enzyme is reduced, inflammatory and oxidative processes promoting Aβ accumulation and formation of toxic oligomers further exacerbating synaptic degeneration (see review ref. 13). As discussed above the findings from the current study and others^10, 16, 17^ do not fully support this notion in human studies, but do not rule out the possibility that IR is associated with early deposition of Aβ.

Two recent studies have shown increases in HOMA-IR associated with increases in Aβ^18, 48^. The study by Willette *et al*.^18^, showed that higher HOMA-IR was associated with higher amyloid load (as assessed by PiB-PET) in normoglycaemic participants recruited from the Wisconsin Registry for Alzheimer’s disease Prevention (WRAP). The study performed analysis of HOMA-IR with PiB-PET and did not include CSF Aβ42, or CSF T-tau/P-tau levels. The more recent study by Hoscheidt *et al*.^48^ assessed participants with a parental history of dementia due to AD recruited from a similar cohort, the Wisconsin Alzheimer’s Disease Research Centre (ADRC), Investigating memory in people at risk, causes and treatments (IMPACT). The study investigated the association of HOMA-IR with CSF AD related biomarkers including Aβ42, CSF sAPPα/sAPPβ and CSF P-tau and showed that increases in HOMA-IR was associated with increased CSF levels of sAPPβ (not sAPPα), and modest association with CSF Aβ42 only. These findings were consistent with IR promoting amyloidogenic processing of APP (i.e. increased sAPPβ) and thus Aβ42 formation. Whilst associations between HOMA-IR and neocortical Aβ burden were not assessed in the latter study, the findings were suggestive of being consistent with that previously observed^18^.

There are number of factors that may account for the differences in the findings in our cohort and the Wisconsin cohorts. Age may have a major role in determining where in the progression of AD pathology, IR has the greatest impact. The participants in the Wisconsin studies were younger (57.7 yrs^49^, and 60 yrs^18^) compared to our study cohort (CN average age 70 yrs.) and other cohorts where no associations with Aβ-amyloid was reported (~75 yrs.^10^ and ~79 yrs.^17^). Therefore, it is possible that an earlier relationship occurs between IR and Aβ-amyloid burden, but in older and presymptomatic individuals a relationship with Tau exists. Longitudinal studies that incorporate Aβ-amyloid imaging, tau imaging and CSF Aβ/tau biomarkers are required to map out where IR fits into the progression of the disease. However, both relationships may be useful in the possible use of IR as a factor that contributes to predicting the conversion to cognitive impairment from pre-symptomatic stages.

It is also worth noting that the Wisconsin cohorts had a high percentage of participants with a family history of dementia due to AD, and in the Hoescheidt *et al*. study^49^, analysis was only performed on those with a family history. In addition, 47% of the cohort were *APOE* ε4 carriers compared to 26.8% in this study and CSF sAPPβ and P-tau/Aβ42 were higher in *APOE* ε4 carriers. The Willette *et al*.^18^ study also had a higher percentage of *APOE* ε4 carriers (38.7%). The carriage of *APOE* ε4 is associated with greater CSF Aβ42 and Aβ-amyloid plaque load^50–53^, a strong predictor of AD pathology and cognitive decline^54, 55^. The apoE4 isoform is also thought to have a greater impact on Aβ accumulation, through impairing clearance/degradation^56–60^, but also has been associated with the dysregulation of APP processing to promote Aβ production^61, 62^. These characteristics of the Wisconsin cohorts may also explain the greater association of HOMA-IR with Aβ-amyloid pathology compared to what we observe in the AIBL cohort.

Our findings, in CN adults, indicate that increases in IR are associated with reductions in cognition function and elevations in CSF tau. This and other studies suggest that IR may contribute to AD progression, although it is possible that it is AD pathology that gives rise to metabolic dysfunction. For example, evidence from AD post-mortem studies shows brain regions involved in regulating brain and systemic energy metabolism, such as the hypothalamus contain Aβ plaques and Tau tangles (see review ref. 63). Further, FDG-PET studies show lowered hypothalamic glucose metabolism in MCI or AD than in age matched controls. Additionally, animal studies suggest that peripheral Aβ may drive IR and glucose dysregulation and that tau may be important in maintaining glucose homeostasis (see review ref. 64). Our findings that IR is associated with preclinical reductions in cognitive performance and increased CSF tau levels contribute to this understanding of the relationship between IR and AD. These data are also consistent with those from a recent cross-sectional study from the ADNI cohort which observed that T2D-related brain atrophy was associated with increases in CSF P-tau in CN, MCI and AD groups and which led to the conclusion that T2D may promote neurodegeneration independent of AD dementia diagnosis^10^. Our observed associations of increasing IR with reductions in cognition and increases in CSF T-tau and P-tau in CN adults suggests IR may also lead directly to changes in cognition and tau accumulation in early AD. Longitudinal analysis, particularly in controls/MCI participants that convert may provide some insight into the direction of this association. Ideally, these studies would be complemented with PET imaging of relevant brain regions including the hypothalamus. Although, FDG-PET studies have shown reduced glucose utilisation in the AD/MCI hypothalamus^65, 66^ studies with Aβ or tau tracers have not been forthcoming, potentially due to the small size of the hypothalamus, limiting spatial resolution, and off-target binding, which may increase the difficulty in assessing this brain region.

There are some limitations to our findings. Firstly, there is the potential that observed findings are currently sample dependent for several reasons; participants were volunteers and not randomly selected from the community, therefore the findings of this study may only be applicable to similar cohorts and the study used cognitive composites derived from specific cognitive assessments, and the use of similar composites derived from different cognitive assessments may result in different findings. Second, the analysis is cross-sectional and informs that there is an association of HOMA indices with cognitive functioning. The findings merit longitudinal analysis to determine if; (1) insulin resistance is associated with the progression of AD pathology and at what stages this relationship is observed (i.e. amyloid at early stages/ tau at later stages) and (2) the contribution of IR/T2D to predicting cognitive impairment. The HOMA indices are calculated from fasting glucose and insulin levels. Although commonly used in association studies, particularly large cohort studies, a closer measure of insulin sensitivity is utilising the euglycaemic-hyperinsulinaemic clamp to quantify insulin secretion and assess metabolism of glucose. Given that this method requires to be done in a clinical setting it would not be feasible in large cohort studies, but once confirmation of a role for IR in the progression of the disease/conversion to cognitive impairment/AD pathology, studies could be undertaken in a small subset to tease out potential mechanisms. As the focus was associations with IR, our study variables did not include cardiovascular or atherosclerotic factors (i.e. cholesterol, triglycerides), however recent studies investigating the relationship of IR with atherosclerotic factors in AD/MCI patients show that increased insulin/decreased response to insulin are independent predictors for AD and MCI^67^.

We have demonstrated that in the AIBL cohort increases in HOMA-IR are associated with reductions in cognitive domains that show early changes in AD with concomitant increases in CSF tau levels. Longitudinal analysis in this and additional cohorts that incorporate tau imaging would be requried to confirm this association with accumnulation of pathological changes. A similar analysis in individuals at risk will also determine if there are earlier associations with Aβ burden and if T2D contributes to the conversion of pre-sympotmatic to early cogntive impairment (MCI) or MCI to AD. As early IR, prior to significant hyperglycaemia, is modifiable this work has implications in identifying and assessing strategies to reduce/prevent the onset cognitive decline and pathology associated with T2D.

## Materials and Methods

### Participants

The study reports on data collected from the AIBL study, a prospective longitudinal study of ageing. This cross-sectional study presents baseline data from 1264 participants (905 cognitively normal (CN), 156 mild cognitive impaired (MCI), 203 Alzheimer’s disease (AD)). Information regarding AIBL study design, enrolment process, neuropsychological assessments and diagnostic criteria has been previously described^68^. Ethics approval for the AIBL study and all experimental protocols was provided by the ethics committees of Austin Health, St Vincent’s Health, Hollywood Private Hospital and Edith Cowan University. All experiments and methods were carried out in accordance with the approved guidelines and regulations and all volunteers gave written informed consent before participating in the study.

### Cognitive Measures

The neuropsychological test battery administered in the AIBL study has been described in detail previously^68^. This included the Mini-Mental State examination (MMSE), Clock drawing test, California Verbal Learning Test- Second edition (CVLT-II), Logical Memory I and II (LMI; LMI; Story A only), D-KEFS verbal fluency, a 30-item version of the Boston Naming Test (BNT), Wechsler Test of Adult Reading (WTAR), Digit Span and Digit Symbol-Coding subtests of the Wechsler Adult Intelligence Scale-Third edition (WAIS-III), the Stroop task (Victoria version), and the Rey Complex Figure Test (RCFT). Cognitive measures are presented as five domain specific composite scores, derived from data collected from the neuropsychological battery as previously published by^69^. The five domain composites included in this study were; Verbal Episodic Memory (VEM; CDR sum of boxes (CDR_SB_), LMII CVLT false positives (CVLT_FP_) and long delay free recall (CVLTLD_FR_)), Visual Episodic Memory (ViEM; CDR_SB_, RCFT; 3 and 30 minute delayed recall, and recognition hits (RCFT_3DR_, RCFT_30DR_ and RCFT_Hits_)), Executive Functioning (EF; CDR_SB_, Stroop, Verbal Fluency Task (FAS), Category Switching Total (CatSwTot)), Language (LANG; CDR_SB_, Category Fluency (CatFl), BNT) and a statistically driven global composite (CDR_SB_, MMSE, LMII, CVLT_FP_ and Clock), the latter aimed as a sensitive measure for longitudinal decline in individuals predisposed to AD^69^. All composites were calculated with a correction for age, sex, years of education, premorbid IQ (WAIS-III Full Scale Intelligence Quotient (FSIQ) and depressive symptoms (Geriatric Depression Scale (GDS))^69^.

### Biochemistry, Genotyping and HOMA estimations

Baseline fasted blood samples were taken and fractionated, with one aliquot sent to clinical pathology laboratories in Perth and Melbourne, as described previously^68^. As part of the clinical pathology assessment, fasting plasma insulin (FPI) and fasting plasma glucose levels (FPG) were assessed. The stated reference ranges are the ranges established in the clinical pathology laboratory in accordance with the national guidelines (http://www.nata.asn.au/, http://www.health.gov.au/npaac). The FPI and FPG were used to calculate values of Homeostatic modelling assessment (HOMA). The HOMA model is often used in cross-sectional and longitudinal studies to estimate insulin sensitivity and pancreatic β-cell functioning as alternatives to more direct assessments such as glucose clamping or acute insulin response, which are not practical in large cohort studies (see review ref. 70). Initially, comparisons of two methods of HOMA was performed; the HOMA1 (“original method”) using equations developed by Mathews and colleagues^71^ and the HOMA2 “the computer model”) developed by Levy and colleagues^72^. These models and the differences between them have been extensively discussed elsewhere^70^. HOMA1 was calculated using the following: HOMA-IR = (FPI x FPG)/22.5; HOMA-B (%) = (20x FPI)/(FPG = 3.5) to estimate IR and β-cell functioning respectively. HOMA2 was calculated from a Microsoft Excel macro accessed via the Oxford University website (www.dtu.ox.ac.uk/homacalculator/). Pearson’s correlation analysis of the data generated from HOMA1 and HOMA2 analysis revealed a strong significant correlation between these indices (HOMA1-IR vs HOMA2-IR, r = 0.976, p < 0.001; HOMA1-B vs HOMA2 B, r = 0.717, p < 0.0001), indicating suitability of both methods for this data set. However, HOMA1 was utilised as it is the most commonly used method in large cohort cross-sectional or longitudinal studies. All reference to HOMA in this report reflects HOMA1 calculated indices.

DNA was extracted and *APOE* genotype determined previously described^73^. Briefly, QIAamp DNA Blood Maxi Kits (Qiagen, Hilden, Germany) were used per manufacturer’s instructions to extract from whole blood. *APOE* genotype was determined from two separate TaqMan® (Thermo Fisher Scientific, Waltham, MA) genotyping assays for the single nucleotide polymoprhisms rs7412 (assay ID: C____904973_10) and rs429358 (assay ID: C___3084793_20). TaqMan® genotyping assays were performed on a QuantStudio 12 K Flex™ Real-Time-PCR systems (Thermo Fisher Scientific, Waltham, MA) using the TaqMan® GTXpress™ Master Mix (Thermo Fisher Scientific, Waltham, MA) methodology as per manufacturer instructions. *APOE* carrier status was defined by the presence (1 or 2 copies; ε4 + ) or absence (0 copies; ε4−) of the *APOE* ε4 allele.

CSF collection and Aβ42, T-tau, and P-tau_181P_ quantitation were performed as previously described^74^. Briefly, approximately 10–14 ml of CSF was collected in the morning by routine lumbar puncture after overnight fasting directly into one 15 ml polypropylene tube (Greiner Bio-One188271), employing a protocol like that recommended by the Alzheimer’s Biomarkers Standardization Initiative^75^. All CSF samples for analysis were taken from aliquots prepared and stored as previously described^74^ and thawed at time of assay. All CSF samples were analyzed in duplicate using the enzyme-linked immunosorbent assay (ELISA): INNOTEST Aβ-amyloid (1–42; Aβ42), INNOTEST hTAU Ag (T-tau), and INNOTEST Phospho-tau (P-tau; 181 P; P-tau181P) (Innogenetics, Ghent, Belgium) per published standard methods. Mean intra-assay coefficients of variation for these assays are as previously published^74^. This study reports on data from 66 study participants from whom CSF was taken at the baseline time point of the AIBL study.

### Brain Imaging

Data was available at baseline from a total of 379 AIBL participants (262 CN, 69 MCI, 48 AD) who underwent Aβ-amyloid imaging with positron emission tomography using either 11C-Pittsburgh Compound B (PiB), 18F-florebetapir or 18F-Flutemetamol as described elsewhere^76–78^. PET standardized uptake value ratios (SUVR) were determined for all tracers using CapAIBL, a web based freely availably MR-less methodology^79^. Briefly, SUVs were summed and normalized to either the cerebellar cortex SUV (PiB), whole cerebellum SUV (florbetapir, FBP) or pons SUV (flutemetamol, FLUTE) to yield the target-region to reference-region SUVR. To allow for the analysis of tracer specific SUVRs as a single continuous variable, a linear regression transformation, termed the “Before the Centiloid Kernel Transformation” (BeCKeT) scale, was applied to FBP and FLUTE SUVR to generate PiB-like SUVR units^80^.

Hippocampal Volume data was available for 319 (229 CN, 52 MCI, 38 AD) participants at baseline. Hippocampal volumes were determined through MRI, parameters of which have been previously described^81^. Briefly, participants underwent T1 weighted MRI using the ADNI 3-dimensional (3D) Magnetization Prepared Rapid Gradient Echo (MPRAGE) sequence on 1.5 T or 3 T scanners. Hippocampal volume was calculated after correcting for age in years and intracranial volume (sum of grey matter, white matter and cerebrospinal fluid volumes), as previously described^82^.

### Statistical analysis

All statistical analysis was conducted using IBM SPSS Statistics (version 23; IBM Corp, Armonk, NY, USA) with the level of significance set to α = 0.05 (two-tailed). All variables were assessed for conformation to a normal distribution. Box-Cox transformations was used to correct variables departing from a normal distribution^83^. For all variables, except HOMA indices, the calculated lambda (λ) equated to no transformation. HOMA indices underwent transformations (T(Y)) prior to analysis, specifically: T(Y) = ((Y + 1)^−0.079^−1)/−0.079 and T(Y) = (Y^0.04^−1)/0.04 for HOMA-IR and HOMA-B, respectively (with Y representing the HOMA index). Analysis of demographic variables was undertaken using a One-way Analysis of Variance (ANOVA) to determine differences in continuous variables across clinical classifications differences in categorical variables determined using the χ^2^-test. Differences in HOMA indices between clinical classifications were assessed using an analysis of covariance (ANCOVA) via a General Linear Model (GLM) with Bonferroni correction. Associations between HOMA indices and cognitive composites and pathological brain changes in CN were assessed using linear regression analysis. Both ANCOVA and linear regression analyses, for all dependent variables, covaried for body mass index (BMI), diabetes (yes/no), diabetes medication (yes/no), hypertension (yes/no), smoking (yes/no), and *APOE* genotype (ε4+/ε4−), with sex and age only covaried for with biomarker dependent variables.

### Data availability

All data and samples used in this study are derived from the Australian Imaging, Biomarkers and Lifestyle (AIBL) Study of Ageing. All AIBL data, and that specific to this study, is publically accessible to all interested parties through an Expression of Interest procedure and is governed by the AIBL Data Use Agreement, for more information please see https://aibl.csiro.au/awd/.
